# Arrhythmia monitoring and outcome after myocardial infarction (BIO|GUARD-MI): a randomized trial

**DOI:** 10.3389/fcvm.2024.1300074

**Published:** 2024-05-13

**Authors:** Christian Jøns, Poul Erik Bloch Thomsen, Sam Riahi, Tom Smilde, Ulrich Bach, Peter Karl Jacobsen, Miloš Táborský, Jozsef Faluközy, Marcus Wiemer, Per Dahl Christensen, Attila Kónyi, Dan Schelfaut, Alan Bulava, Marcin Grabowski, Béla Merkely, Dieter Nuyens, Rajiv Mahajan, Patrick Nagel, Roland Tilz, Jerzy Malczynski, Clemens Steinwender, Johannes Brachmann, Harvey Serota, Jürgen Schrader, Steffen Behrens, Peter Søgaard

**Affiliations:** ^1^ Department of Cardiology, Rigshospitalet, University of Copenhagen, Copenhagen, Denmark; ^2^ Department of Cardiology, Aalborg University Hospital, Aalborg, Denmark; ^3^ Department of Cardiology, Scheperziekenhuis, Treant Zorggroep, Emmen, Netherlands; ^4^ Department of Cardiology, Vivantes Humboldt-Klinikum, Berlin, Germany; ^5^ Department of Cardiology, Fakultní Nemocnice Olomouc, Olomouc, Czech Republic; ^6^ National Hospital of Cardiology, Balatonfüred, Hungary; ^7^ Department of Cardiology, Johannes Wesling Universitätsklinik, Minden, Germany; ^8^ Department of Cardiology, Viborg Regional Hospital, Viborg, Denmark; ^9^ Heart Institute, The University of Pécs, Pécs, Hungary; ^10^ Cardiovascular Centre, Onze Lieve Vrouw Clinic Aalst, Aalst, Belgium; ^11^ Department of Cardiology, České Budějovice Hospital and Faculty of Health and Social Sciences, University of South Bohemia, České Budějovice, Czech Republic; ^12^ First Department of Cardiology, Medical University of Warsaw, Warsaw, Poland; ^13^ Heart and Vascular Centre, Semmelweis University, Budapest, Hungary; ^14^ Ziekenhuis Oost-Limburg, Genk, Belgium; ^15^ Department of Cardiology, Lyell McEwin Hospital, and Adelaide Medical School, The University of Adelaide, Adelaide, SA, Australia; ^16^ Department of Cardiology, Charité Universitätsmedizin Berlin, Berlin, Germany; ^17^ Department of Cardiology, Universitätsklinikum Schleswig-Holstein - Campus Lübeck, Lübeck, Germany; ^18^ Herning Hospital, Herning, Denmark; ^19^ Department of Cardiology, Kepler University Hospital Linz, Linz, Austria; ^20^ Department of Cardiology, Klinikum Coburg, Coburg, Germany; ^21^ Department of Cardiology, St. Louis Heart and Vascular, Bridgeton, MO, United States; ^22^ Biotronik SE & Co. KG, Berlin, Germany; ^23^ Department of Cardiology, Vivantes Humboldt-Klinikum and Klinikum Spandau, Berlin, Germany

**Keywords:** cardiac arrhythmia, myocardial infarction, implantable cardiac monitor, telemedicine, randomized controlled trial

## Abstract

**Objectives:**

Cardiac arrhythmias predict poor outcome after myocardial infarction (MI). We studied if arrhythmia monitoring with an insertable cardiac monitor (ICM) can improve treatment and outcome.

**Design:**

BIO|GUARD-MI was a randomized, international open-label study with blinded outcome assessment.

**Setting:**

Tertiary care facilities monitored the arrhythmias, while the follow-up remained with primary care physicians.

**Participants:**

Patients after ST-elevation (STEMI) or non-ST-elevation MI with an ejection fraction >35% and a CHA_2_DS_2_-VASc score ≥4 (men) or ≥5 (women).

**Interventions:**

Patients were randomly assigned to receive or not receive an ICM in addition to standard post-MI treatment. Device-detected arrhythmias triggered immediate guideline recommended therapy changes via remote monitoring.

**Main outcome measures:**

MACE, defined as a composite of cardiovascular death or acute unscheduled hospitalization for cardiovascular causes.

**Results:**

790 patients (mean age 71 years, 72% male, 51% non-STEMI) of planned 1,400 pts were enrolled and followed for a median of 31.6 months. At 2 years, 39.4% of the device group and 6.7% of the control group had their therapy adapted for an arrhythmia [hazard ratio (HR) = 5.9, *P* < 0.0001]. Most frequent arrhythmias were atrial fibrillation, pauses and bradycardia. The use of an ICM did not improve outcome in the entire cohort (HR = 0.84, 95%-CI: 0.65–1.10; *P* = 0.21). In secondary analysis, a statistically significant interaction of the type of infarction suggests a benefit in the pre-specified non-STEMI subgroup. Risk factor analysis indicates that this may be connected to the higher incidence of MACE in patients with non-STEMI.

**Conclusions:**

The burden of asymptomatic but actionable arrhythmias is large in post-infarction patients. However, arrhythmia monitoring with an ICM did not improve outcome in the entire cohort. Post-hoc analysis suggests that it may be beneficial in non-STEMI patients or other high-risk subgroups.

**Clinical Trial Registration:**

[https://www.clinicaltrials.gov/ct2/show/NCT02341534], NCT02341534.

## Strengths of this study

• Our study investigates the effect of continuous monitoring of arrhythmias in post-MI patients
• Because the target population is large and their risk of acute deteriorations is high, a clinical benefit might be very meaningful
• The methods are clinically applicable

## Limitations of this study

• Blinding was impossible and unbiased collection of clinical events was challenging

## Introduction

Ischemic heart disease (IHD) is a leading cause of death and disability worldwide (1). Patients with chronic IHD are under a considerable risk to experience major adverse cardiac events (MACE) such as myocardial (re-) infarction, stroke, or acute worsening of heart failure. After cardiac rehabilitation, bi-annual evaluations by a general practitioner or cardiologist are recommended in the first year (2), but follow-up may be too infrequent to detect an acute deterioration of the patient's status and thereby prevent future events. In patients with an implantable cardioverter-defibrillator (ICD), continuous remote monitoring of cardiac arrhythmias shortens the time to clinical decision and subsequently improves prognosis in some studies (3–7), though results are conflicting (8).

The CARISMA and SMART-MI trials have shown that post-infarction patients without an indication for an ICD suffer from a significant burden of arrhythmias as documented using an implantable cardiac monitor (ICM) (9, 10). In CARISMA, asymptomatic arrhythmias preceeded MACE in the majority of patients (9, 11, 12). However, no randomized trial has tested whether monitoring and responding to arrhythmias detected on an ICD improves prognosis in high-risk post-infarction patients.

In the present randomized controlled trial, we compared the clinical outcome of standard treatment without ICM against an ICM-based treatment with remote monitoring in high-risk patients after myocardial infarction (MI). The hypothesis was that a fast response to ICM-detected arrhythmias using a predefined investigational algorithm to guide diagnosis and treatment would result in a reduction a combined endpoint of cardiovascular death and hospitalization (13).

## Material and methods

### Study design

BIO|GUARD-MI (Biomonitoring in Patients with Preserved Left Ventricular Function after Diagnosed Myocardial Infarction) was an international, randomized, parallel group, open-label study with a blinded outcome assessment and an event-driven design. The investigational sites were 60 tertiary clinical centers in Europe, the USA, and Australia (see Supplemental Material). Details of the trial design have been published previously (13). A Steering Committee developed the study concept, designed and oversaw the study, had full access to all data, supervised the analysis, and had ﬁnal responsibility for the decision to submit for publication. An independent Data and Safety Monitoring Board (DSMB) guided the study (Supplemental Material).

### Patients

Patients were enrolled if they had a history of MI and a CHA_2_DS_2_-VASc score ≥4 (men) or ≥5 (women). Although originally constructed to estimate the risk of stroke, we used the CHA_2_DS_2_-VASc score as a simple tool to identify patients with a higher risk for primary endpoints and thereby reduce the number of required patients (13). Further, a left ventricular ejection fraction >35% was required excluding patients eligible for an implantable cardioverter/defibrillator (ICD). Patients with oral anticoagulation therapy, Parkinson's disease, hemorrhagic diathesis, chronic renal dialysis, or a cardiac implantable electronic device were excluded (13).

### Randomization and masking

The patients were randomly assigned in a 1:1 ratio to receive an ICM with remote monitoring (ICM group) or no ICM (control group), in addition to standard post-MI treatment. The randomization was generated through a centralized, concealed process implemented by the sponsor and stratiﬁed by site and by the presence (STEMI) or absence (NSTEMI) of ST-segment elevation at the index infarction. Neither patients nor investigators were blinded to the group assignment, because a sham implantation of an inactive device was considered too risky and inconvenient for control group patients.

### Procedures

Patients randomized to the ICM group received a BioMonitor 2-AF or BIOMONITOR III device (Biotronik SE & Co. KG, Germany) (14, 15). After subcutaneous insertion, the ICM records 1-minute ECGs for atrial fibrillation, bradycardia, high ventricular rate, or asystole episodes and automatically transmits up to six ECGs per day to the secure Biotronik Home Monitoring website (16). The suggested device programming is available in the design paper (13).

ICM-detected ECGs were screened every working day by the Central ECG Monitoring Board (see Supplemental Material). ECGs that were not caused by over- or undersensing were entered into the study database. Investigators were required, within 7 days, to adjudicate the rhythm, decide on the clinical relevance, and report whether they would contact the patient. Decisions about subsequent changes of therapy were in the responsibility of the investigator who followed prespecified recommendations based on current guidelines (13). The investigator implemented them or delegated them to the patient's general practitioner or cardiologist.

Because follow-up in tertiary care centers is not standard of care in post-MI patients, study subjects were advised to undergo regular follow-up by their general practitioner or cardiologist, according to local standard post-MI practice. Because ICMs do not need regular follow-up by the device clinic (in contrast to therapeutic devices such as pacemakers or ICDs), no study visits were scheduled in either arm. Patients were explicitly instructed not to visit or contact the investigational site on their own initiative, but to refer to their personal physician in case of symptoms. Investigators contacted patients only after arrhythmia detection by the ICM. No contacts were required to manage remote monitoring transmissions.

The lack of regular follow-up visits in the investigational site made adverse event reporting challenging. All patients were called by telephone every 6 months by an interviewer of a contract research organization who asked them about adverse events from the preceding period. These interviewers did not interfere in the medical treatment or give advice in any way. If the patient reported any hospitalization or other medical occurrence, the study investigators were informed. The interviewer also supported the provision of discharge letters, death certificates or other documentation of the event. For every adverse event, it was reported whether an arrhythmia was involved that required a change in therapy according to current guidelines (13).

### Outcomes

The primary endpoint was MACE, defined as a composite of cardiovascular death, urgent visit for acute worsening of heart failure, or acute unscheduled hospitalization due to arrhythmia, acute coronary syndrome, heart failure, stroke, systemic embolism, or major bleeding (included to capture side effects of anticoagulation treatment). Events resulting from ICM detection were considered scheduled and were not counted as endpoints. An independent blinded Endpoint and Adverse Event Committee adjudicated all adverse events that fulfilled predefined criteria. The major secondary endpoint was the time to the first arrhythmia that resulted in a therapeutic change. Further secondary endpoints were all-cause mortality, components of the composite primary endpoint, the time to the first arrhythmia, and quality of life.

### Adverse event reporting bias and premature termination

Based on the unblinded results of the first interim analysis, the DSMB detected an increased incidence of adverse events in the ICM group. They assumed that this was probably attributable to a reporting bias resulting from the unblinded trial design and informed the steering committee, although the formal futility criterion was not fulfilled. A small unblinded team of sponsor personnel involved external independent experts to judge whether a bias in the clinical event reporting existed and to suggest corrective measures. The quality of adverse events reporting was compared between the study groups using the time to the first *non-cardiovascular* adverse event because ICMs should not modify this event type. A significantly lower non-cardiovascular adverse event rate in the control group was found. Based on the fact that the group allocation was randomized and the assumption that the ICM cannot cause non-cardiovascular events, this result was interpreted as evidence of a more complete adverse event reporting in ICM patients. This bias appeared possible because ICM group patients knew of their device and may have seen reasons to contact the study site, even if they were explicitly asked not to do this; this could result in a higher chance of medical events getting attention of the investigational site staff. If a bias of the same amplitude was present also in primary endpoints, it was judged large enough to inevitably cause a negative study result.

Patient enrollment was stopped while follow-up continued. Efforts to get reports of all adverse events in both study groups were stepped up by re-training of the telephone interviewers, by additional telephone contacts to patients and by intensified source data verification. While these corrective measures reduced the bias, they did not resolve it completely after a considerable investment of time and effort. Thus, a protocol amendment was introduced which defined that all patients were to be invited to the investigational site and asked about all hospitalizations during the study, and then to exit the study. The trial was prematurely terminated after the last patient visit.

In the final data, the difference in the time to the first non-cardiovascular adverse events was not statistically significant between the study groups (HR = 1.08; *P* = 0.38). Thus, the analysis presented here assumes unbiased reporting. This assumption is conservative, since a hypothetical remaining bias would reduce the study effect.

### Statistical analysis

It was initially planned to enroll and follow patients until 372 primary endpoints had occurred, with interim analyses after 124 and 248 primary events (a 3-stage adaptive group sequential test procedure according to O'Brian Fleming) (13). This gave an 80% power given an assumed hazard ratio (HR) of 0.7452. However, the group sequential design was abandoned because of the unblinding of limited sponsor personnel after the first interim analysis. The modified plan in the amended protocol was a final analysis based on all available data after premature study termination.

The primary hypothesis was tested with the log rank test stratified for NSTEMI/STEMI in all patients with follow-up, based on the intention-to-treat principle.

If non-fatal outcomes are frequent, the exclusion of recurrent events by the Kaplan-Meier method reduces the power to detect outcome differences (17). To compensate for the power lost due to the premature study termination, a post-hoc analysis including recurrent events was conducted using the Andersen-Gill method with robust estimates for the standard errors. The influence of predefined subgroups on the study effect was also tested including recurrent events.

Missing data were not imputed. Other time-to-event data than the primary endpoint were analyzed with the Cox-regression model and visualized with the Kaplan-Meier method.

To test post-hoc whether the apparent study benefit in the NSTEMI subgroup depends on the higher risk of NSTEMI patients, we used the multivariate Cox method and identified baseline variables linked with a higher incidence of the primary endpoint, including recurrent events. This was done in the control group because study treatment can modify the risk. As input variables, we selected all available baseline characteristics and comorbidities that affected between 10% and 90% of patients, except the STEMI/NSTEMI variable. Few missing values were imputed with the more frequent value. We tested whether the number of risk factors per patient showed statistical interaction with the study effect.

Quantitative variables are described as mean ± SD or as median and interquartile range (IQR). Continuous and ordinal data were compared with the non-parametric Mann–Whitney-Wilcoxon test, and nominal data with the exact Fisher test. A two-sided *P*-value <0.05 was considered statistically significant. All analyses were performed with SAS 9.4.

## Results

### Study population

**Figure 1:**
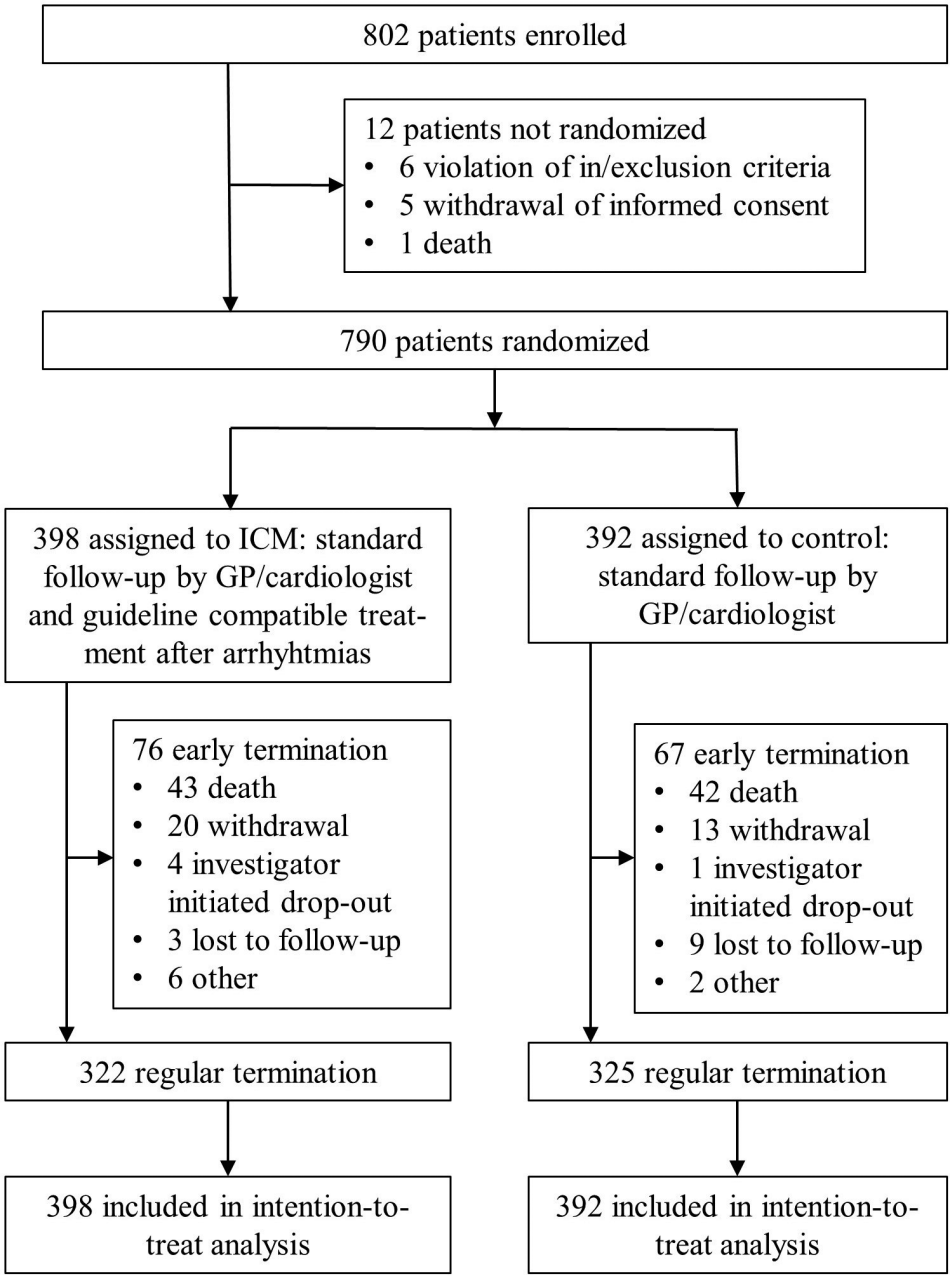
Trial profile. GP, general practitioner; ICM, implantable cardiac monitor.

Between August 2015 and April 2020, 802 patients were enrolled, of whom 790 were randomly assigned (Figure 1): 398 to the ICM group, 392 to the control group. Patient characteristics were well balanced between the two groups (Table 1). Mean age was 71.2 years (SD 8.1), and 568 (72%) patients were men. In the ICM group, 366 (92%) patients received an ICM, and in the control group, one patient mistakenly received an ICM; all patients were analysed in the group to which they were randomized. Of 790 randomized patients, 647 (82%) completed follow-up, 85 died (11%), and 58 (7%) terminated the study prematurely (Figure 1).

**Table 1:** Characteristics of the intention-to-treat population at baseline^a^.

Characteristic	ICM group (*N* = 398)	Control group (*N* = 392)
Male gender	291 (73.1%)	277 (70.7%)
Age (years)	71.5 (7.7)	70.8 (8.5)
Body mass index (kg/m^2^)^b^	29.3 (5.6)	29.2 (4.8)
Number of MIs before the index MI		
0	332 (83.4%)	322 (82.1%)
1	42 (10.6%)	57 (12.6%)
2	14 (3.5%)	9 (2.3%)
3	9 (2.3%)	4 (1.0%)
4	1 (0.3%)	0 (0.0%)
PCI done at the index MI	314 (78.9%)	321 (81.9%)
Type of the index MI		
NSTEMI	205 (51.5%)	198 (50.5%)
STEMI	193 (48.5%)	194 (49.5%)
Time between the index MI and enrollment		
≤ 40 days	137/395 (34.7%)	141/388 (36.3%)
> 40 days	258/395 (65.3%)	247/388 (63.7%)
LVEF (%)	52.8 (8.5)	53.0 (8.4)
CHA_2_DS_2_-VASc score^c^	4.8 (0.9)	5.0 (1.0)
Congestive heart failure	135 (33.9%)	136 (34.7%)
NYHA functional class	1.72 (0.62)	1.76 (0.68)
Diabetes	240 (60.3%)	244 (62.2%)
Stroke, TIA, or other thromboembolic event	88 (22.1%)	109 (27.8%)
Stroke	52 (13.1%)	66 (16.8%)
TIA	38 (9.5%)	36 (9.2%)
Other thromboembolic event	5 (1.3%)	10 (2.6%)
Peripheral arterial disease	54 (13.6%)	42 (10.7%)
Chronic obstructive pulmonary disease	40 (10.4%)	47 (12.6%)
Kidney failure	49 (12.7%)	40 (10.4%)
Medication		
Beta blocker	334 (83.9%)	318 (81.1%)
ACEi/ARB	323 (81.2%)	301 (76.8%)
Calcium antagonist	153 (38.4%)	129 (32.9%)
Diuretic	160 (40.2%)	145 (37.0%)
Aldosterone blocker	42 (10.6%)	45 (11.5%)
Statin	359 (90.2%)	347 (88.5%)
Class III antiarrhythmic drug	11 (2.8%)	10 (2.6%)
Anticoagulant	39 (9.8%)	50 (12.8%)
Platelet aggregation inhibitor	369 (92.7%)	361 (92.1%)

Data are mean (SD), *n* (%), or n/N (%).

ACEi, angiotensin-converting enzyme inhibitors; ARB, angiotensin receptor blockers; LVEF, left ventricular ejection fraction; MI, myocardial infarction; NSTEMI, no ST-segment elevation MI; NYHA, New York Heart Association; PCI, percutaneous coronary intervention; STEMI, ST-segment elevation MI; TIA, transient ischemic attack.

^a^ No baseline variable differed between groups, even without correction for multiple testing.

^b^ Body mass index is the weight in kilograms divided by the square of the height in meters.

^c^ The score includes **C**ongestive heart failure (1 point), **H**ypertension (1 point), **A**ge ≥75 years (2 points), **D**iabetes (1 point), previous **S**troke or transient ischemic attack (2 points), **V**ascular disease (1 point, e.g., myocardial infarction), **A**ge 65–74 years (1 point), and female **S**ex **c**ategory (1 point).

The median length of follow-up was 31.6 months (IQR 21.8–45.4). Cumulative follow-up was 1,071 vs. 1,120 patient-years (ICM vs. control group).

### Detection of arrhythmias and treatment

**Figure 2:**
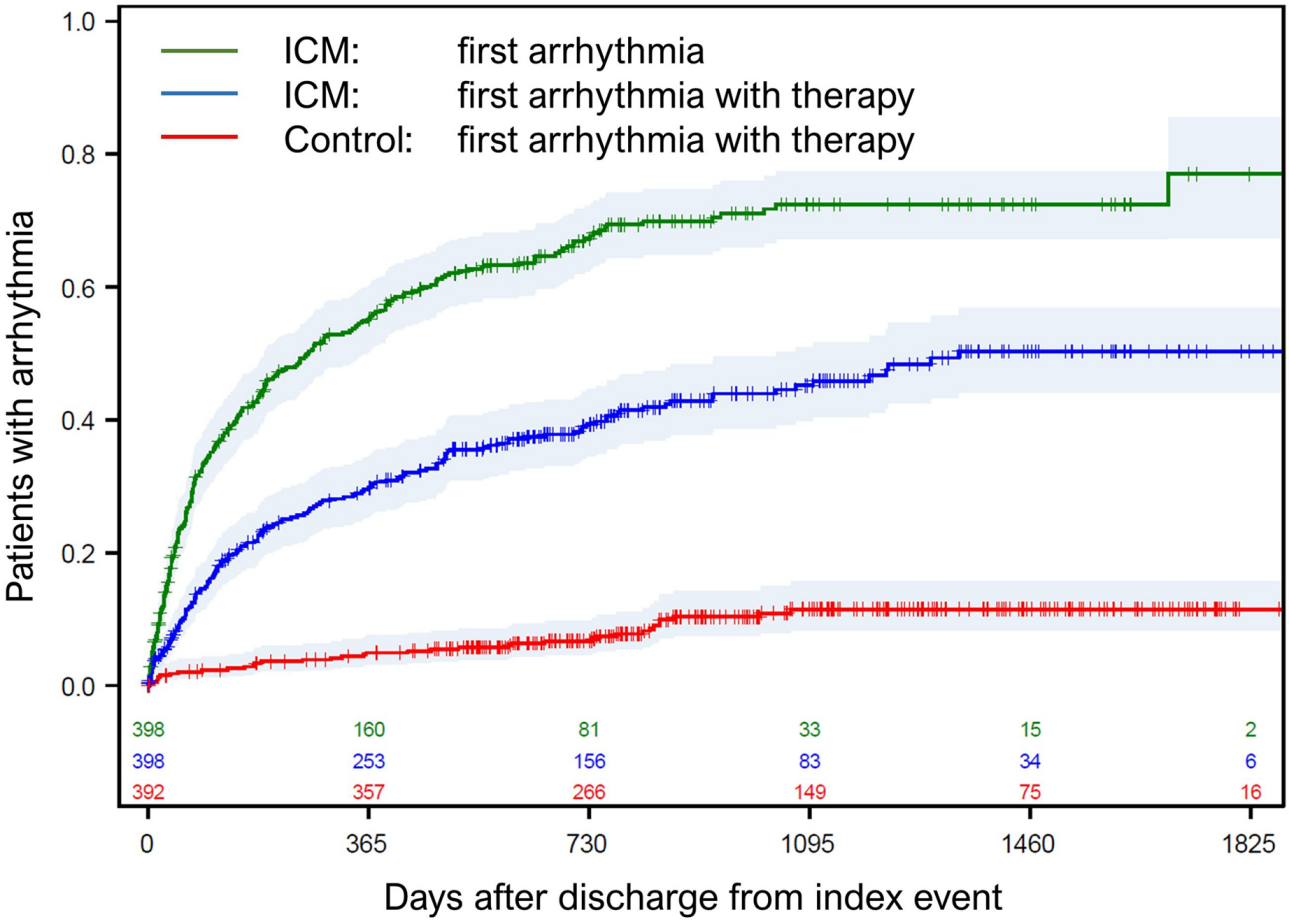
Time to first arrhythmia. Time to first ICM-detected arrhythmia (green line), time to first arrhythmia resulting in a therapy change in the ICM group (blue line), and time to first arrhythmia resulting in a therapy change in the control group (red line). Day 0 is the discharge from index hospitalization. ICM, implantable cardiac monitor.

Of 2,548 arrhythmia notifications that were sent by the Central ECG Monitoring Board, 1,909 were classified as true arrhythmias. Divided by the study duration in the ICM group, this results in 1.8 true arrhythmias per patient-year. Most frequent were bradycardic events (48.4%, including sinus bradycardia, pause, and atrioventricular block) and atrial fibrillation (33.0%) (Table 2). For 482 arrhythmia notifications, a contact to the patient was planned. The median delay between arrhythmia and patient contact was 4 days (IQR 2–6 days). At 2 years, 67.3% of the ICM group had a first arrhythmia and 39.4% received a guideline required change in therapy, as compared to 6.7% of control patients (HR = 5.9, *P* < 0.0001; Figure 2). The number of adverse events with arrhythmias leading to therapy modification was 213 in the ICM group (affecting 161 patients) and 50 in the control group (36 patients). Within these adverse events, the most frequent arrhythmias were atrial fibrillation (*N* = 120), leading to oral anticoagulation initiation in 79.2% of cases, and bradycardic events (*N* = 103), leading to a pacemaker implantation (49.5%) or reduction of heart rate lowering drugs (45.6%). Less frequent were other supraventricular arrhythmias (*N* = 19) or ventricular tachycardias (*N* = 17). Overall, 11 implantable cardioverter-defibrillators and six catheter ablations were indicated.

**Table 2:** ECG recordings sent to the investigational site.

ECG rhythm classification by the investigators	*N*=	% of true arrhythmia
Total	2,548	
Noise/artifact or uninterpretable	255	
Normofrequent sinus rhythm	343	
Sinus tachycardia	41	
True arrhythmia (investigator classification)	1,909	100%
Atrial fibrillation	629	33.0%
Bradycardia	593	31.1%
Pause	228	11.9%
Non-sustained ventricular tachycardia	129	6.8%
Other supraventricular tachyarrhythmia	124	6.5%
Atrioventricular block	103	5.4%
Frequent ventricular premature beats	71	3.7%
Atrial flutter	24	1.3%
Sustained ventricular tachyarrhythmia	8	0.4%
Polymorphic ventricular tachycardia or fibrillation	0	0%

The percentage of patients with oral anticoagulation treatment was increased at study termination compared to baseline in both study groups, but more in the ICM group (37.5%) than in the control group (25.1%; *P* = 0.0007). In NSTEMI patients, the use of ACEi/ARB at study termination was higher in the ICM group than in the control group (75.4% vs. 62.5%; *P* = 0.013), but in STEMI patients it was similar in both groups (74.4% vs. 70.8%; *P* = 0.47).

### Clinical outcome

#### Primary result

**Figure 3:**
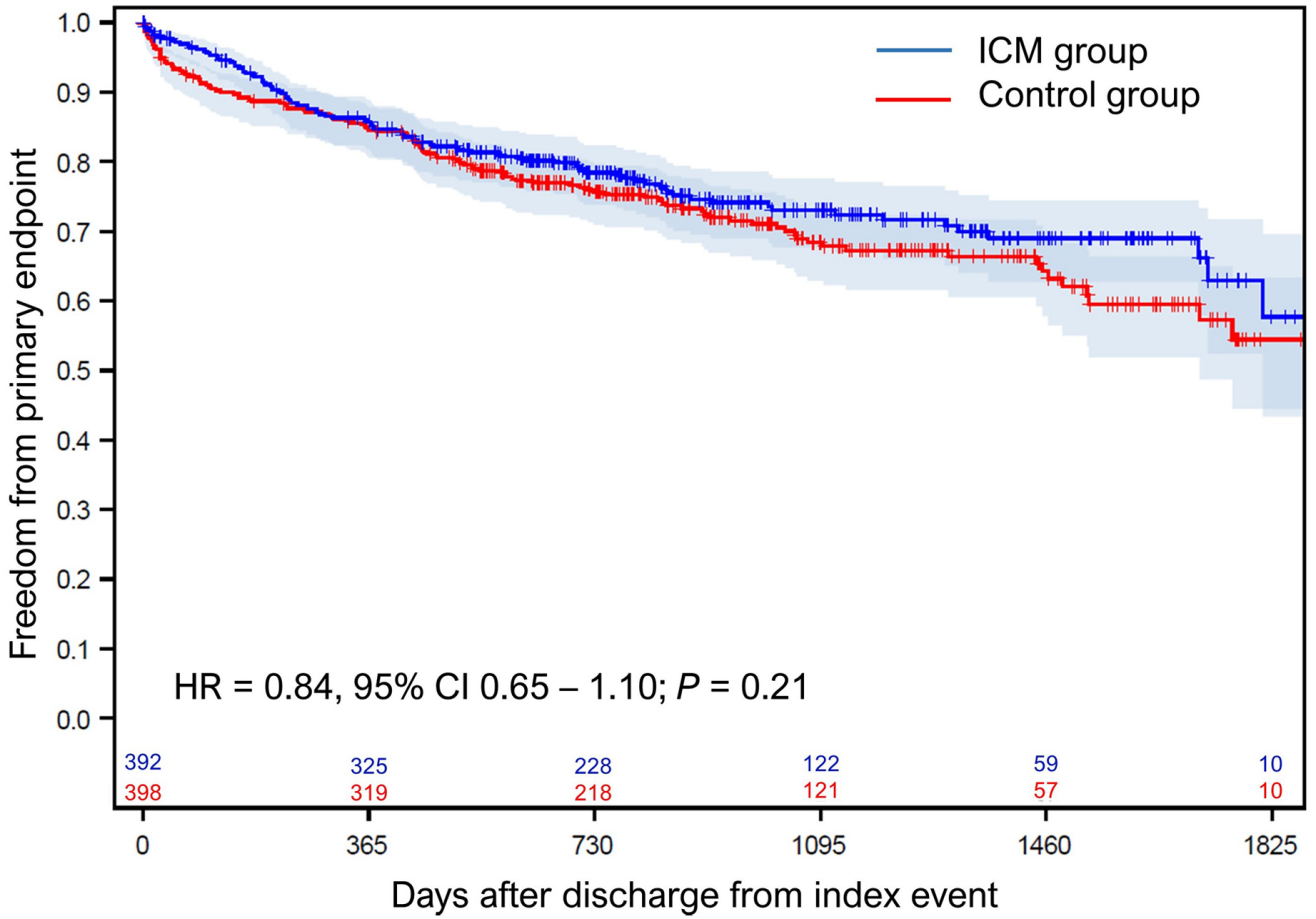
Freedom from primary endpoint. The probability of freedom from primary endpoint. Day 0 is the discharge from index hospitalization. CI, confidence interval; HR, hazard ratio; ICM, implantable cardiac monitor.

A total of 336 primary endpoint events occurred in 218 patients. There was no statistically significant difference in primary endpoints between the study groups (HR = 0.84, 95%-CI 0.65–1.10; *P* = 0.21; Figure 3). Most frequent endpoints were hospitalizations for acute coronary syndrome and for worsening of heart failure (Table 3).

**Table 3:** Patients with primary endpoints.

Event	All patients			STEMI subgroup		NSTEMI subgroup	
	Total (*N* = 790)	ICM group (*N* = 398)	Control group (*N* = 392)	ICM group (*N* = 193)	Control group (*N* = 194)	ICM group (*N* = 205)	Control group (*N* = 198)
	Patients (events)	Patients (events)	Patients (events)	Patients (events)	Patients (events)	Patients (events)	Patients (events)
Primary endpoint	218 (336)	99 (143)	119 (193)	43 (66)	42 (58)	56 (77)	77 (135)
Death	43 (43)	20 (20)	23 (23)	10 (10)	8 (8)	10 (10)	15 (15)
CV death	28 (28)	15 (15)	13 (13)	6 (6)	5 (5)	9 (9)	8 (8)
Unclear reason	15 (15)	5 (5)	10 (10)	4 (4)	3 (3)	1 (1)	7 (7)
Any hosp.	202 (312)	93 (134)	109 (178)	40 (60)	36 (52)	53 (74)	73 (126)
Reason for hosp.							
Acute CS	120 (160)	48 (65)	72 (95)	20 (29)	25 (36)	28 (36)	47 (59)
MI	68 (85)	31 (40)	37 (45)	14 (19)	16 (18)	17 (21)	21 (27)
Unstable angina	61 (75)	21 (25)	40 (50)	7 (10)	14 (18)	14 (15)	26 (32)
Worsening HF	52 (75)	25 (32)	27 (43)	10 (14)	5 (6)	15 (18)	22 (37)
Arrhythmia	31 (32)	15 (16)	16 (16)	7 (8)	3 (3)	8 (8)	13 (13)
Stroke	33 (34)	19 (19)	14 (15)	8 (8)	5 (5)	11 (11)	9 (10)
Major bleeding	7 (8)	1 (1)	6 (7)	0 (0)	2 (2)	1 (1)	4 (5)
Systemic embolism	3 (3)	1 (1)	2 (2)	1 (1)	0 (0)	0 (0)	2 (2)
Urgent visit for heart failure	0 (0)	0 (0)	0 (0)	0 (0)	0 (0)	0 (0)	0 (0)

Data represent number of patients with event, and data in parantheses represent number of events. A patient who had hospital admission and death was counted in both categories but was a single patient in the total endpoint number.

CS, coronary syndrome; CV, cardiovascular; HF, heart failure; hosp., hospitalization; ICM, implantable cardiac monitor; MI, myocardial infarction; NSTEMI, absence of ST-segment elevation at the index myocardial infarction; STEMI, presence of ST-segment elevation.

#### *Post-hoc* analyses and subgroups

The *post-hoc* analysis including recurrent events according to Andersen-Gill utilized all 336 primary endpoint events from 218 patients. It confirmed the Kaplan-Meier result of no significant benefit (HR = 0.77, 95%-CI 0.57–1.03).

**Figure 4:**
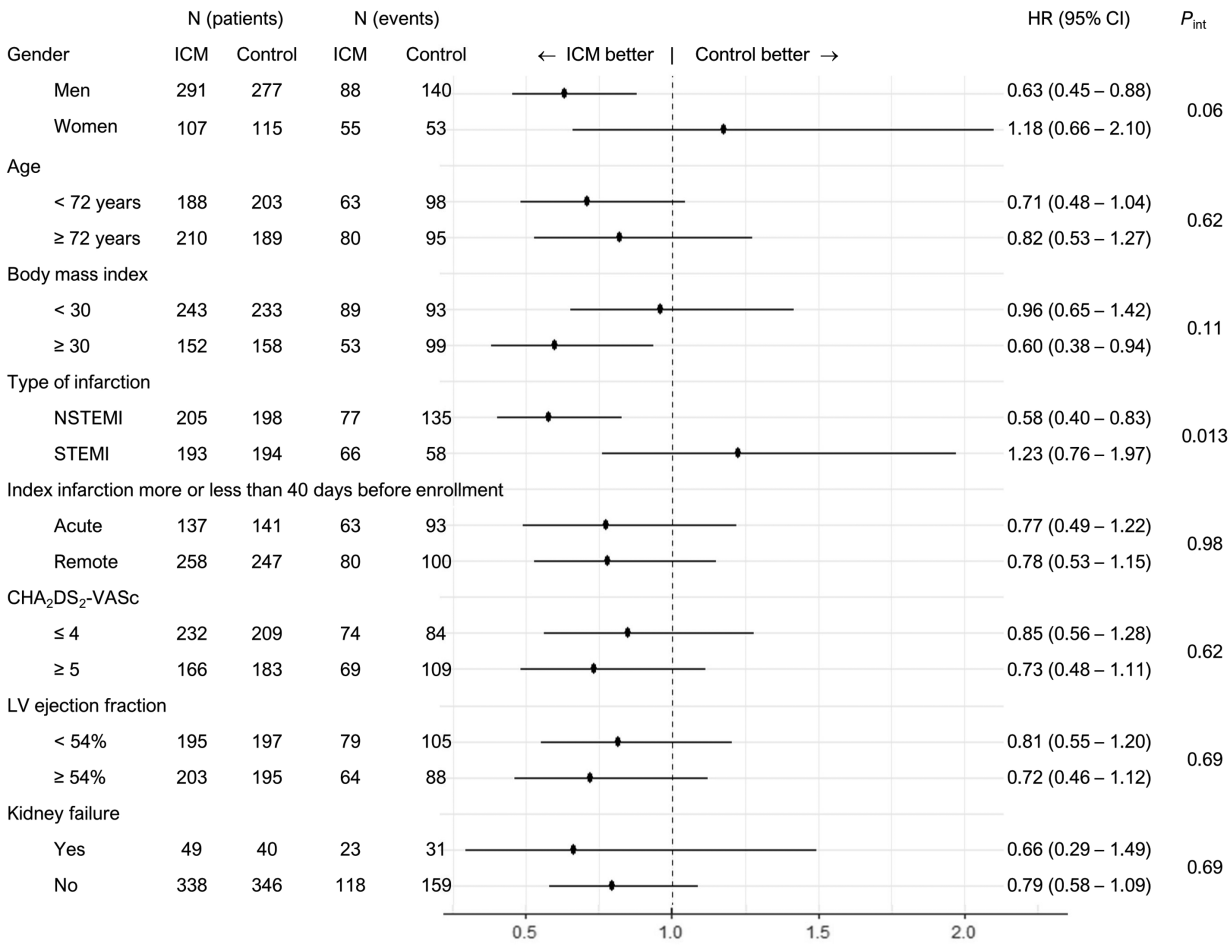
Predefined subgroup analyses of the primary endpoint. Only STEMI/NSTEMI showed significant interaction with the study effect. Subgroups with a study benefit were NSTEMI, male gender and BMI >30. For the definition of CHA_2_DS_2_-VASc, see Table 1. CI, confidence interval; HR, hazard ratio; ICM, implantable cardiac monitor group; Int, interaction; LV, left ventricular; NSTEMI, absence of ST-segment elevation; STEMI, presence of ST-segment elevation at the index myocardial infarction.

Of the prespecified subgroups, only the STEMI/NSTEMI variable showed significant interaction with the study effect (Figure 4). Patients with NSTEMI (*N* = 403; HR = 0.58, 95%-CI 0.40–0.83) appeared to have a benefit but patients with STEMI (*N* = 387; HR = 1.23; 95%-CI 0.76–1.97) did not (*P*_interaction_ = 0.013).

The incidence of primary endpoints was higher in the NSTEMI than STEMI subgroup of patients (HR = 1.78, *P* = 0.0002). To investigate whether the apparent benefit in the NSTEMI subgroup depends on the higher risk in these patients, we constructed a risk model. The included variables and their influence on the incidence of the primary endpoint in the control group are shown in Table 4. In multivariate analysis, we excluded the NSTEMI / STEMI variable because we were interested in the effect of other risk factors than this variable. Four variables remained as predictors: peripheral artery disease, kidney failure, body mass index >30 and MI within 40 days of enrollment. The number of risk factors per patient exerted a borderline significant effect on the study benefit (*P*_interaction_ = 0.051), with a higher number of risk factors connected to a higher study benefit.

**Table 4:** Baseline variables linked with a higher incidence of primary endpoint, including recurrent events.

Baseline variable (control group, *N* = 392)	PE Events/Patients with risk factor	PE Events/Patients without risk factor	Univariate model		Multivariate model		
			HR	*P*-value	HR	95% CI	*P*-value
NSTEMI	135/198	58/194	2.51	<0.0001			
Peripheral artery disease	38/42	155/350	2.37	0.0004	2.26	1.36–3.76	0.0016
Kidney failure	31/40	162/352	1.89	0.047	1.64	0.84–3.19	0.14
COPD	30/47	163/345	1.50	0.17			
Body mass index >30	99/158	94/234	1.47	0.060	1.49	1.01–2.22	0.047
MI ≤40 days of enrollment	93/141	100/251	1.46	0.069	1.39	0.94–2.07	0.01
Stroke/TIA/TE	64/109	129/283	1.26	0.27			
Congestive heart failure	78/136	115/256	1.19	0.42			
Age >median (72 years)	95/189	98/203	1.19	0.41			
LVEF < median (54%)	88/197	105/195	1.14	0.53			
Diabetes	124/244	69/148	1.11	0.63			
Female gender	53/115	140/277	0.95	0.84			

COPD, chronic obstructive pulmonary disease; HR, hazard ratio; LVEF, left ventricular ejection fraction; MI, myocardial infarction; NSTEMI, absence of ST-segment elevation at the index MI; PE, primary endpoint; TE, thromboembolic event; TIA, transient ischemic attack.

## Discussion

In post-MI patients with a CHA_2_DS_2_-VASc score ≥4 (≥5 in women), continuous arrhythmia monitoring with an ICM leads to a considerable amount of guideline-directed therapy changes. The BIO|GUARD–MI trial confirms similar findings of CARISMA (9) and SMART-MI (10), but in contrast to these trials, it is the only trial so far designed to test if responding clinically to these arrhythmias has a clinical effect. Our study was terminated early due to difficulties with the unbiased collection of adverse events; the final analysis failed to prove that guideline-directed study treatments provide a clinical benefit in the total study population.

The inclusion of repeated events in patients who survived the first MACE provides a more accurate estimate of the effect size (17). This analyses included 336 events, close to the prespecified 372 events, and confirmed the findings of the protocol defined analysis.

The analysis of predefined subgroups found that the type of index infarction (NSTEMI or STEMI) had a significant interaction with the study effect (*P*_interaction_ = 0.013), with a large risk reduction in NSTEMI patients, but no effect in STEMI patients. A comparison of STEMI and NSTEMI patients showed that the incidence of MACE was by far higher in NSTEMI than in STEMI patients, an observation that has been described before (18, 19). Patients with NSTEMI did not display a systematic difference in baseline characteristics compared to STEMI patients. The added post-hoc multivariate risk model using all available baseline variables except whether the index MI was STEMI or NSTEMI showed that the number of risk factors interacted with the study effect with borderline statistical significance. A higher risk of meeting an endpoint was connected to a higher benefit of monitoring by the ICM. Hence, the negative findings of the BIO|GUARD-MI trial are possibly related to a too low clinical risk of the included population. Whether there may be a benefit in patients with higher risk, e.g., the NSTEMI population or other subgroups will need further analysis.

The most frequent treatments were oral anticoagulation after atrial fibrillation and reduction of beta blockade or pacemaker implantation after bradycardia or pauses. Despite this we observed the strongest trends (no differences were statistically significant) in reductions of acute coronary syndrome and worsening of heart failure whereas strokes were not reduced. However, since 37% of ICM patients vs. 25% of non-ICM patients were on anticoagulations at the end of the study, these event numbers may be due to chance. CARISMA found a very strong correlation between ICM detected pauses and cardiovascular death (HR 6.6, *P* < 0.001), many of which were due to terminal heart failure (9). This influence is also not obvious. It may be possible that arrhythmias detected by an ICM serve as risk indicators of a worsening status, after which immediate action is required, rather than a trigger of a major cardiac event. Higher-risk patients may simply require more attention, which is triggered by arrhythmia detection in the ICM group, and they may have more therapeutic options after arrhythmia diagnosis. We found a higher share of NSTEMI patients with ACEi/ARB at termination in the ICM group than in the control group, suggesting that the treatment of patients after arrhythmias may improve prognostically effective therapies which are not related to the arrhythmia. The recently concluded SMART-MI study had a design comparable to our trial but failed to show even a trend towards improvement in the secondary clinical outcome endpoint (10). This may be due to an insufficient sample size, but also due to a better-than-normal post-MI patient care with 6-monthly follow-ups in tertiary centers, possibly leaving less room for improvements after arrhythmias were detected.

### Limitations

Our study must be interpreted considering several limitations. First, we have discontinued the study prematurely after a planned interim analysis raised doubt about the integrity of the adverse event and endpoint reporting. The concern about the validity of results from studies with premature termination is that termination may be intentional at a time when interim results are favorable. We can rule this out because the termination (after the last patient's final visit) was defined in an amended study protocol more than half a year before study closure. Second, while procedural changes allowed us to establish reporting of adverse events without evident bias, we did not meet the targeted number of primary endpoints. This increases the likelihood of a false-negative study result but does not put the results in doubt. Third, although the NSTEMI/STEMI subgroups were prespecified, the absence of a statistically significant study effect in the total population precludes a precise estimation of the alpha error in subsequent subgroup analyses. However, the interaction of risk factors derived from a multivariate analysis with the study benefit suggests that the increased risk in the NSTEMI subgroup may be the basis of the observed difference between NSTEMI and STEMI subgroups.

## Conclusion

In conclusion, post-MI patients with mildly reduced or preserved ejection fraction have a large burden of asymptomatic arrhythmias. Arrhythmia monitoring with an ICM allows the implementation of guideline recommended therapies in consequence of these arrhythmias. Our study failed to show that this strategy improves the outcome in the total study population. Sub-analyses indicate that the strategy could be beneficial in patient populations at higher risk for cardiovascular events and should encourage further research in the field of arrhythmia monitoring, and more generally the monitoring of physiological parameters, in chronically diseased patients.

## Data Availability

The raw data supporting the conclusions of this article will be made available by the authors, without undue reservation.

## References

[1] Khan MA Hashim MJ Mustafa H Baniyas MY Al Suwaidi SKBM AlKatheeri R Global epidemiology of ischemic heart disease: results from the global burden of disease study. Cureus. (2020) 12(7):e9349. 10.7759/cureus.9349 32742886 PMC7384703

[2] Knuuti J Wijns W Saraste A Capodanno D Barbato E Funck-Brentano C 2019 ESC guidelines for the diagnosis and management of chronic coronary syndromes. Eur Heart J. (2020) 41:407–77. 10.1093/eurheartj/ehz425 31504439

[3] Hindricks G Taborsky M Glikson M Heinrich U Schumacher B Katz A Implant-based multiparameter telemonitoring of patients with heart failure (IN-TIME): a randomised controlled trial. Lancet. (2014) 384:583–90. 10.1016/S0140-6736(14)61176-4 25131977

[4] Crossley GH Boyle A Vitense H Chang Y Mead RH. The CONNECT (clinical evaluation of remote notification to reduce time to clinical decision) trial: the value of wireless remote monitoring with automatic clinician alerts. J Am Coll Cardiol. (2011) 57:1181–9. 10.1016/j.jacc.2010.12.012 21255955

[5] Guédon-Moreau L Lacroix D Sadoul N Clémenty J Kouakam C Hermida JS A randomized study of remote follow-up of implantable cardioverter defibrillators: safety and efficacy report of the ECOST trial. Eur Heart J. (2013) 34:605–14. 10.1093/eurheartj/ehs425 23242192 PMC3578267

[6] Hindricks G Varma N Kacet S Lewalter T Søgaard P Guédon-Moreau L Daily remote monitoring of implantable cardioverter-defibrillators: insights from the pooled patient-level data from three randomized controlled trials (IN-TIME, ECOST, TRUST). Eur Heart J. (2017) 38:1749–55. 10.1093/eurheartj/ehx015 29688304 PMC5461472

[7] Geller JC Lewalter T Bruun NE Taborsky M Bode F Nielsen JC Implant-based multi-parameter telemonitoring of patients with heart failure and a defibrillator with vs. Without cardiac resynchronization therapy option: a subanalysis of the IN-TIME trial. Clin Res Cardiol. (2019) 108:1117–27. 10.1007/s00392-019-01447-5 30874886 PMC6753058

[8] Morgan JM Kitt S Gill J McComb JM Ng GA Raftery J Remote management of heart failure using implantable electronic devices. Eur Heart J. (2017) 38:2352–60. 10.1093/eurheartj/ehx227 28575235 PMC5837548

[9] Bloch Thomsen PE Jons C Raatikainen MJ Moerch Joergensen R Hartikainen J Virtanen V Long-term recording of cardiac arrhythmias with an implantable cardiac monitor in patients with reduced ejection fraction after acute myocardial infarction: the cardiac arrhythmias and risk stratification after acute myocardial infarction (CARISMA) study. Circulation. (2010) 122:1258–64. 10.1161/CIRCULATIONAHA.109.902148 20837897

[10] Bauer A Sappler N von Stülpnagel L Klemm M Schreinlechner M Wenner F Telemedical cardiac risk assessment by implantable cardiac monitors in patients after myocardial infarction with autonomic dysfunction (SMART-MI-DZHK9): a prospective investigator-initiated, randomised, multicentre, open-label, diagnostic trial. Lancet Digit Health. (2022) 4:e105–16. 10.1016/S2589-7500(21)00253-3 35090674

[11] Jons C Jacobsen UG Joergensen RM Olsen NT Dixen U Johannessen A The incidence and prognostic significance of new-onset atrial fibrillation in patients with acute myocardial infarction and left ventricular systolic dysfunction: a CARISMA substudy. Heart Rhythm. (2011) 8:342–8. 10.1016/j.hrthm.2010.09.090 21093611

[12] Gang UJ Jøns C Jørgensen RM Abildstrøm SZ Messier MD Haarbo J Risk markers of late high-degree atrioventricular block in patients with left ventricular dysfunction after an acute myocardial infarction: a CARISMA substudy. Europace. (2011) 13:1471–7. 10.1093/europace/eur165 21665919

[13] Jons C Sogaard P Behrens S Schrader J Mrosk S Bloch Thomsen PE. The clinical effect of arrhythmia monitoring after myocardial infarction (BIO-GUARD|MI): study protocol for a randomized controlled trial. Trials. (2019) 20:563. 10.1186/s13063-019-3644-5 31511057 PMC6737710

[14] Piorkowski C Busch M Nölker G Schmitt J Roithinger FX Young G Clinical evaluation of a small implantable cardiac monitor with a long sensing vector. Pacing Clin Electrophysiol. (2019) 42:1038–46. 10.1111/pace.13728 31119745 PMC6851891

[15] Deneke T Cabanas P Hofer D Gaspar T Pierre B Bisignani G New-generation miniaturized insertable cardiac monitor with a long sensing vector: insertion procedure, sensing performance, and home monitoring transmission success in a real-world population. Heart Rhythm O2. (2022) 3:152–9. 10.1016/j.hroo.2022.01.010 35496450 PMC9043386

[16] Søgaard P Behrens S Konyi A Taborsky M Christiansen PD Jacobsen PK Transmission and loss of ECG snapshots: remote monitoring in implantable cardiac monitors. J Electrocardiol. (2019) 56:24–8. 10.1016/j.jelectrocard.2019.06.005 31233982

[17] Rogers JK Pocock SJ McMurray JJ Granger CB Michelson EL Östergren J Analysing recurrent hospitalizations in heart failure: a review of statistical methodology, with application to CHARM-preserved. Eur J Heart Fail. (2014) 16:33–40. 10.1002/ejhf.29 24453096 PMC4822681

[18] Polonski L Gasior M Gierlotka M Osadnik T Kalarus Z Trusz-Gluza M A comparison of ST elevation versus non-ST elevation myocardial infarction outcomes in a large registry database: are non-ST myocardial infarctions associated with worse long-term prognoses? Int J Cardiol. (2011) 152:70–7. 10.1016/j.ijcard.2010.07.008 20684999

[19] Vora AN Wang TY Hellkamp AS Thomas L Henry TD Goyal A Differences in short- and long-term outcomes among older patients with ST-elevation versus non-ST-elevation myocardial infarction with angiographically proven coronary artery disease. Circ Cardiovasc Qual Outcomes. (2016) 9:513–22. 10.1161/CIRCOUTCOMES.115.002312 27601458

