# Supplementary material for: Arrhythmia monitoring and outcome after myocardial infarction (BIO|GUARD-MI): a randomized trial
Source: Front Cardiovasc Med. 2024 May 13;11:1300074. doi: 10.3389/fcvm.2024.1300074 (PMC11132184; doi:10.3389/fcvm.2024.1300074)
Supplement: Supplementary file 1 [file Datasheet1.docx]

**Supplemental Material**

Supplement to: Jøns C, et al. “Arrhythmia monitoring and outcome after myocardial infarction (BIO|GUARD-MI): a randomized trial”

**Contents: Detailed Methods Page**

Investigational Sites ..………………………………………………………………….. 2

Committees and Boards ……………………………………………………………….. 5

Contract Research Organization ………………………………………………………. 5

**Investigational Sites**

Countries are listed alphabetically, with the number of randomized patients per country (N=). Investigational sites are ordered according to patient contribution, and investigators are listed alphabetically within three categories: principal investigator (PI), co-investigators (Co-Inv.), and assistants.

**Australia (N=19, 2.4% of the study cohort):**

- R. Mahajan (PI), M. A. Arstall, A. Chan, D. Lypourlis, G. Mahadavan, D. A. Munawar, P. Pati, A. Philpott (Co-Inv.), A. Garrett, B. Hoffmann, J. Koch, G. McMichael, J. Rose (Lyell McEwin Hospital (LMH), Elizabeth Vale)
- N. Stoyanov (PI), G. Hillis, S. C. Lee, H.-W. A. Yau (Co-Inv.), M. Bonner, C. Dias, L. Hilllis, K. Ireland (Royal Perth Hospital, Perth)
- R. Pathak (PI), O. Krepysheva, M. Laco, A. Rana, N. Siddon, P. Taverner, P. York (The Canberra Hospital, Canberra)

**Austria (N=11, 1.4%):**

- C. Steinwender (PI), A. Fellner, S. Hönig, A. Kypta, K. A. Saleh, S. Schwarz (Co-Inv.), N. Jungmayr, E.-M. Lambrakis, S. Schabetsberger (Kepler University Hospital, Linz)

**Belgium (N=50, 6.3%):**

- D. Schelfaut (PI), S. Chatzikyriakou, T. J. R. De Potter, P. Geelen, P. Peytchev (Co-Inv.), S. Aerts, H. Batjoens, K. De Knijf, H. Delacroix, H. Descamps, W. Timmermans, K. Van Bockstal, P. Wouter (Onze Lieve Vrouw Clinic Aalst, Aalst)
- D. Nuyens (PI), J. Dens, M. Rivero-Ayerza, H. Van Herendael (Co-Inv.), W. Helsen, N. Peeters, C. Swijsen, E. Theunissen, V. Vangeel, F. Vermeulen (Ziekenhuis Oost-Limburg, Genk)

**Czech Republic (N=62, 7.8%):**

- M. Táborský (PI), V. Doupal, M. Fedorco, V. Gloger, M. Vicha (Co-Inv.), P. Kohoutkovà, I. Opavská, E. Soucková (Fakultní nemocnice Olomouc, Olomouc)
- K. Sedláček, H. Wünschová (PI), J. Kohoutek, L. Krýže, M. Levčík, M. Segeťová (Co-Inv.), K. Havlikova, M. Krausová, A. Kuprova, T. Novakova, A. Polednova (Institute for Clinical and Experimental Medicine (IKEM), Praha)
- A. Bulava (PI), P. Hájek, O. Ošmera (Co-Inv.), A. Novotný (České Budějovice Hospital, České Budějovice)
- L. Křivan (PI), M. Kozák, M. Sepši (Co-Inv.) (University Hospital Brno, Brno)

**Denmark (N=168, 21.3%):**

- P. Sogaard (PI), K. M. Al-Zuhairi, F. Heath, S. P. Hjortshøj, K. Holm, G. Klausz, J. M. Larsen, J. G. Rasmussen, J. Ravkilde, S. Riahi, A. M. Thøgersen (Co-Inv.), M. Becker, M. Bøgeskov Søttrup, A. Fladkjar Pedersen, C. Schmidt Skov, K. Villefrance (Aalborg University Hospital, Aalborg)
- J. C. Nielsen (PI), C. Gerdes, H. K. Jensen, R. Kirkfeldt, J. Kristensen, S. B. Kristiansen, M. B. Kronborg, J. Nielsen (Co-Inv.), K. Andersen, S. Runge (Århus University Hospital, Århus N)
- J. Malczynski (PI), M. Böttcher (Co-Inv.), M. Andersen, V. Lynggaard, S. Sondergaard, L. Stampe (Herning Hospital, Herning)
- J. Brock Johansen (PI), T. Olsen, J. Pontoppidan, N. Sandgaard (Co-Inv.), H. Cappelen, T. Gents, M. Rønn, I. Rosenlund, H. Tveskov (Odense University Hospital, Odense)
- P. Dahl Christensen (PI), A. E. Albertsen, J. Refsgaard, D. Svenstrup Moller, J. K. Wilhjelm (Co-Inv.), K. Andersen, H. Bundgaard, G. B. Eriksen, S. Gudmundsdottir, C. Jespersen, P. Saugmann (Regionshospitalet Viborg, Viborg)
- P. K. Jacobsen (PI), C. Jøns, N. Risum (Co-Inv.), M. C. J. Larsen, M. Tarras-Wahlberg, B. H. Thomsen (Rigshospitalet, University of Copenhagen, Copenhagen​)
- T. M. Melchior (PI), O. Dyg, K. Haugan (Co-Inv.), P. Fogh, P. Hansen, S. E. Heinsvig, I. Larsen, V. Perret-Gentil, S. Truesen (Sjaellands University Hospital, Roskilde, Roskilde)

**France (N=9, 1.1%):**

- B. Pierre (PI), D. Angoulvant, D. Babuty, T. Genet, F. Ivanes, C. Saint Etienne (Co-Inv.), D. Bomia, C. Piat, C. Verne (CHRU de Tours, Chambray-lès-Tours)
- R. Eschalier (PI), R. Bosle, P.-A. Catalan, F. Jean (Co-Inv.), L. Cubizolles, A. Thalamy (Hôpital Gabriel Montpied, Clermont Ferrand, Clermont-Ferrand)

**Germany (N=156, 19.7%):**

- P. Nagel, M. J. Roser (PI), P. J. Attanasio, B. Bellmann, S. Biewener, A. Heuberger, M. Huemer, B. Juri, A.-S. Schatz, S. Suhail Arain, V. Tscholl (Co-Inv.), K. Hubert, S. Kuehn, A. Maiwald, I. Redlich, E. Roldan, V. Tauckert (Charité Universitätsklinikum - Campus Benjamin Franklin, Berlin)
- M. Busch (PI), E. Abdiu, A. Bachmann, M. Frenzel, A. Krüger (Co-Inv.), D. Kniephoff (Ernst-Moritz-Arndt-Universität Greifswald, Greifswald)
- G. Hindricks (PI), K. Bode, M. Döring, S. Hilbert, M. Kühl, J. Lucas, S.-L. Richter, P. Sommer (Co-Inv.), B. Fritzsche, D. Günther, J. Koch, H. Winkler (Herzzentrum Leipzig GmbH, Leipzig)
- M. Wiemer (PI), I. Barndt, S. Dürrwald, A. Samol (Co-Inv.), L. Tiedemann, H. S. Wuttig (Johannes Wesling Universitätsklinikum Minden, Minden)
- J. Brachmann (PI), I. Ajmi, S. Busch, M. Held, C. Mahnkopf, T. Mischke, A. Saleh, S. Schnupp, C. Schwab, Z. Vejnovic (Co-Inv.), P. Denninger, A. Höhn, M. Jesse, S. Rube, A. Scharmentke, L. Schwarzmann (Klinikum Coburg, Coburg)
- L. Vitali-Serdoz (PI), D. Bastian, J. Walaschek (Co-Inv.), G. Eichinger (Klinikum Fürth, Fürth)
- T. Deneke (PI), E. Ene, P. M. Halbfaß, K. Nentwich, K. Sonne (Co-Inv.), L. Benkert, O. Gabriel, A. Gans, S. Gaul, D. Gerlach, A.-K. Hein, M. Jahn, S. Schüssler, K. Zeiger (Rhön-Klinikum Campus Bad Neustadt , Bad Neustadt a.d. Saale)
- W. Jung (PI), J. Kohler, B. Roggenbuck-Schwilk, G. Sadeghzadeh (Co-Inv.), M. Lehrer, O. Ruff, L. Wacker (Schwarzwald-Baar Klinikum Villingen-Schwenningen GmbH, Villingen-Schwenningen)
- M. Winterhalter (PI), J. Hoffmann, B. Mollenhauer, S. Purle, I. Ratzmann, A. Schmid, N. Seidel-Mukdessi, A. Voigt, T. Vokrri, A. Walter (Co-Inv.), M. Michaelis, R. Pfaff (SRH Wald-Klinikum Gera GmbH, Gera)
- C. Stellbrink (PI), B. Hansky, C. Köster, D. Meyer zu Vilsendorf (Co-Inv.), B. Brettschneider, M. Iselt (Städtisches Krankenhaus Bielefeld-Mitte, Bielefeld)
- R. Surber (PI), A. Große, J. Känel, F. Walther (Co-Inv.), S. Grund, R. Hariri, S. Springer (Universitätsklinikum Jena, Jena)
- R. Tilz (PI), B. Brüggemann, C. Eitel, T. Fink, J. Vogler (Co-Inv.), C. Breithaupt, A. Gassmann, A. Maaß, K. U. Petersen, A. Rieck (Universitätsklinikum Schleswig-Holstein (UKSH) - Campus Lübeck, Lübeck)
- P. J. Nordbeck (PI), O. Maniuc, M. Moritz (Co-Inv.), C. Moser (Universitätsklinikum Würzburg, Würzburg)
- P. Zierock (PI), H. Seibt (Co-Inv.), B. Eisenhardt, M. Gregor (Vivantes-Krankenhaus Spandau, Berlin)
- S. Behrens (PI), U. Bach, J. Ebbinghaus (Co-Inv.), M. Gregor, V. D. Guillot, S. Johannsen, R. Sartipi, A. Ullmann (Vivantes Humboldt-Klinikum, Berlin)
- J. C. Geller (PI), M. Frommhold, M. Schreiber (Co-Inv.), A. Hasch, R. Meiland (Zentralklinik Bad Berka GmbH, Bad Berka)

**Hungary (N=109, 13.8%):**

- C. Földesi (PI), T. Breuer, A. Kardos, Z. Nagy, Z. Som (Co-Inv.), E. Fülöp (Gottsegen National Cardiovascular Center, Budapest)
- G. Duray (PI), Z. Bari, P. Bogyi, K. Kósa (Co-Inv.), S. Baranyai, T. Fonád (Hungarian Defence Forces Military Hospital, Budapest)
- J. Faluközy (PI), D. Aradi, G. Harmati, K. Harsányi, C. Jenet, B. Kelemen, G. Veress (Co-Inv.), M. V. Hovány (National Hospital of Cardiology, Balatonfüred)
- B. Merkely (PI), O. Kiss, L. Molnár, R. Papp, P. Perge (Co-Inv.), B. Déri, A. Hermecz, C. Lakatos, J. Lovistyék, Z. Majoros, A. Nagy, M. Szabó (Heart and Vascular Center, Semmelweis University, Budapest)
- Z. Csanádi (PI), C. Jenei, A. Kiss, A. Leny, E. Nagy-Baló, S. Sipka, R. Urbancsek (Co-Inv.), V. J. Szatmari (The Debrecen University of Medicine, Debrecen)
- A. Kónyi (PI), M. Németh (Co-Inv.), M. Kulcsár (Heart Institute, The University of Pécs, Pécs)

**Latvia (N=21, 2.7%):**

- A. Erglis (PI), I. Ansaberga, J. Ansabergs, M. Blumbergs, N. Nesterovics, M. Vikmane (Co-Inv.) (Pauls Stradins Clinical University Hospital, Riga)
- J. Verbovenko (PI), J. Pudulis (Co-Inv.) (Riga East Clinical University Hospital, Riga)

**Netherlands (N=80, 10.1%):**

- M. Khan (PI), G. S. De Ruiter, T. Oomens, A. Paes, M. Scheffer, A. Thakur, A. van Staaveren, P. Visser, M. Werner (Co-Inv.), H. Bakker, F. Bosman, J. Happe, R. Turan (Onze Lieve Vrouwe Gasthuis Amsterdam, Amsterdam)
- T. Smilde (PI), M. de Bie, M. de Haart, R. J. M. de Vries, J. P. Edel, E. Hoekstra, L. Kleijn, M. Oosterga, G. Sahin, J. Santman, M.-L. van der Wielen, R. van Es, T. Vet, R. Vijn (Co-Inv.), H. Giezen, D. Ketelaar, J. Krikken, L. Schaafsma, M. Schreurs, S. van der Kooi (Scheperziekenhuis, Treant Zorggroep, Emmen)

**Poland (N=28, 3.5%):**

- M. Grabowski (PI), Ł. Januszkiewicz, D. Paskudzka (Co-Inv.) (Klinika i Katedra Chorób Wewn. i Kardiologii, Warszawa)
- M. Sterliński (PI), A. Oreziak, E. Świerzyńska, A. Witowicz (Co-Inv.), E. Jakubowska, K. Krajewska-Piestrzyńska, E. Nowacka, J. Starosz, M. Włodarczyk (The National Cardiology Institute of Stefan Cardinal Wyszynski - National Research Institute, Warszawa)

**Slovakia (N=14, 1.8%):**

- J. Sedlák (PI), M. Gbur, B. Kafkova, P. Murin (Co-Inv.) (East-Slovak Cardiology Institute (VUSCH), Košice)
- I. Rybar (PI), S. Sivakova (Co-Inv.) (SÚSCCH, Banska Bystrica)

**Spain (N=27, 3.4%):**

- F. Alfonso (PI), P. Antuña, T. Bastante, J. Cuesta, M. Garcia, F. Rivero (Co-Inv.) (Hospital de la Princesa, Madrid)
- J. Martí Almor (PI), B. Benito Villabriga, B. Casteigt, J. I. Jiménez López, E. Valles (Co-Inv.), P. Cabero, C. Soler (Hospital del Mar, Barcelona)
- S. Del Prado (PI), G. L. Alonso, D. Cordero Pereda, E. Franco (Co-Inv.), T. Casado, P. Gonzalez (Hospital Universitario Ramón y Cajal, Madrid)

**USA (N=36, 4.6%):**

- R. Borge (PI), C. Gottlieb, B. Klugherz, V. Mallavarapu (Co-Inv.), G. McCarthy (Abington Medical Specialists, Abington)
- J. Go (PI), R. Aboufakher, K. Olson (Co-Inv.), K. P. Chow, B. Westacott, A. Wirkus (Altru Health System, Grand Forks)
- P. Singh (PI), T. Baki, J. Brown, M. Dhokai, N. Mori, V. Patel, M. Reyer, T. Shah, J. Shah, S. Singhi, M. White (Co-Inv.), J. Bryant, K. Thibault (Carolina Cardiology Associates, Rock Hill)
- V. Shah (PI), M. Ciminelli, J. Elias, P. Slota (Co-Inv.), E. Ghaly (Carolina Heart Specialists, LLC, Lancaster)
- N. Assi (PI), Y. Abdulnabi, T. Al-Joundi, B. Al-Joundi, M. Logue, M. Walter, L. Younis (Co-Inv.), A. Hamud-Socoro (Gateway Cardiology, St. Louis)
- M. Foster (PI), T. Ballard, R. Mehta, S. Miller, S. Reed, M. Sharma (Co-Inv.), N. Polakiewicz (Metro Knoxville HMA LLC, Knoxville)
- H. Serota (PI), P. Chandra, D. Glascock, S. Kalvaitis, R. Ramadan, M. Tahir, G. Vardi (Co-Inv.), P. Brown, S. Miner (St. Louis Heart and Vascular, Bridgeton)
- S. Rosero (PI), M. Aktas, J. Bisognano, J. Gallagher, B. Hall, M. Hamer (Co-Inv.), H. Kopin (University of Rochester, Rochester)

**Committees and Boards**

**Steering Committee:** Christian Jøns (Copenhagen, Denmark), Poul Erik Bloch Thomsen (Aalborg, Denmark), Peter Søgaard (Aalborg, Denmark), Steffen Behrens (Berlin, Germany). The committee was responsible for developing and monitoring the implementation of the study protocol and for ensuring timely publication of results.

**Data and Safety Monitoring Board:** John Camm (London, United Kingdom), Walter Lehmacher (Köln, Germany), Gerhard Steinbeck (Starnberg, Germany). The board regularly reviewed accumulating study data to address patient safety and ethical issues of the study, and it performed the interim analysis and the final analysis of the primary endpoint. Based on the interim data, the responsibility of the board was to request to stop the clinical investigation for superiority or to give a recommendation to the steering committee and the sponsor whether to continue the clinical investigation as planned, to adapt the sample size, or to stop the clinical investigation for safety reasons or for futility. The board members did not participate in the study as investigators.

**Endpoint and Adverse Event Committee:** Ralf Birkemeyer (Oberndorf, Germany), Hubertus Degen (Neuss, Germany), Rainer Halfenberg (Werne, Germany), Michael Oeff (Brandenburg, Germany). The committee analyzed adverse events with respect to the specified endpoint criteria and adjudicated if a primary or secondary endpoint was met. Documentation of clinical events that was not in English or German language was translated into English by a qualified translation service. The committee members did not participate in the study as investigators and were unaware of the treatment group assignment. Every event was adjudicated independently by three members of the board. Cases of disagreement were resolved by discussion in regular meetings.

**Central ECG Monitoring Board:** Judith Piorkowski (Dresden, Germany) and Sandra Leß (Dresden, Germany). The board received alerting e-mails from the Home Monitoring Service Center and reviewed all transmitted ECGs. It discarded detections of noise or artifacts. When an event fulfilled predefined criteria, the responsible physician was notified per e-mail. At the same time, the board entered the event in the study database to ensure correct recording of the event, including the precise time of the ECG transmission, needed to evaluate the time to the investigator’s reaction. The board also provided a centralized follow-up on the investigator’s assessment. The investigator was responsible for checking and adjudicating the arrhythmias immediately but at the latest within 7 days after receiving an arrhythmia notice from the board. The board was not involved in ECG interpretation and medical decisions.

**Contract Research Organization**

**Contract research organization conducting the regular phone calls:** Alexander Neumer, Marcel Kunadt, Pia Bach (Institut für Herzinfarktforschung, Ludwigshafen, Germany). The organization contacted the patients early after enrolment, and then regularly every 6 months. They completed quality-of-life questionnaires and interviewed patients whether they had experienced adverse events in the preceding period. If the patients reported such events, the organization informed the responsible investigator and supported him or her with procuring required documentation for endpoint adjudication, such as discharge letters.
